# Enoyl-CoA hydratase mediates polyhydroxyalkanoate mobilization in *Haloferax mediterranei*

**DOI:** 10.1038/srep24015

**Published:** 2016-04-07

**Authors:** Guiming Liu, Shuangfeng Cai, Jing Hou, Dahe Zhao, Jing Han, Jian Zhou, Hua Xiang

**Affiliations:** 1 State Key Laboratory of Microbial Resources, Institute of Microbiology, Chinese Academy of Sciences, Beijing, China; 2 CAS Key Laboratory of Microbial Physiological and Metabolic Engineering, Institute of Microbiology, Chinese Academy of Sciences, Beijing, China; 3 University of Chinese Academy of Sciences, Beijing, China

## Abstract

Although polyhydroxyalkanoate (PHA) accumulation and mobilization are one of the most general mechanisms for haloarchaea to adapt to the hypersaline environments with changeable carbon sources, the PHA mobilization pathways are still not clear for any haloarchaea. In this study, the functions of five putative (*R*)-specific enoyl-CoA hydratases (R-ECHs) in *Haloferax mediterranei*, named PhaJ1 to PhaJ5, respectively, were thoroughly investigated. Through gene deletion and complementation, we demonstrated that only certain of these ECHs had a slight contribution to poly(3-hydroxybutyrate-*co*-3-hydroxyvalerate) (PHBV) biosynthesis. But significantly, PhaJ1, the only R-ECH that is associated with PHA granules, was shown to be involved in PHA mobilization in this haloarchaeon. PhaJ1 catalyzes the dehydration of (*R*)-3-hydroxyacyl-CoA, the common product of PHA degradation, to enoyl-CoA, the intermediate of the β-oxidation cycle, thus could link PHA mobilization to β-oxidation pathway in *H. mediterranei*. This linkage was further indicated from the up-regulation of the key genes of β-oxidation under the PHA mobilization condition, as well as the obvious inhibition of PHA degradation upon inhibition of the β-oxidation pathway. Interestingly, 96% of *phaJ*-containing haloarchaeal species possess both *phaC* (encoding PHA synthase) and the full set genes of β-oxidation, implying that the mobilization of carbon storage in PHA through the β-oxidation cycle would be general in haloarchaea.

Enoyl coenzyme A (enoyl-CoA) hydratases (ECHs) reversibly catalyze the *syn* and *anti* hydration of 2-enoyl-CoA to produce (*S*)- or (*R*)-3-hydroxyacyl-CoA (3HA-CoA)^1^. The (*S*)-specific ECHs (S-ECHs) are involved in fatty acid β-oxidation^2^. Through catalyzing the hydration of intermediates in fatty acid β-oxidation, the (*R*)-specific ECHs (R-ECHs) may play an important role in fatty acid metabolism in eukaryotes^3^ and in polyhydroxyalkanoate (PHA) biosynthesis in bacteria^4^,^5^,^6^.

PHAs accumulate in granular form and serve as storage resources of carbon and energy in many bacteria^7^ and archaea^8^ under conditions of carbon excess. The PhaJ_Ac_ in *Aeromonas caviae* was the first identified R-ECH supplying monomers for PHA biosynthesis^4^,^9^. The gene of PhaJ_Ac_ forms a cluster with the genes of PhaP (phasin) and PhaC (PHA synthase) in the organization of *phaP*-*phaC*-*phaJ*^9^. PhaJ_Ac_ catalyzes the hydration of enoyl-CoA from the fatty acid β-oxidation pathway and provides monomers of C_4_-C_6_ chain length for PHA biosynthesis^4^. PhaJ_Ac_ forms a homodimer with the monomer catalytic dyad that is formed by Asp_31_ and His_36_, and the residues Leu_65_ and Val_130_ define the chain length of the substrates^10^,^11^. Four *phaJ* genes (*phaJ1*_*Pa*_ to *phaJ4*_*Pa*_) were identified in the bacterium *Pseudomonas aeruginosa*^5^,^12^. Among the four PhaJs, PhaJ1_Pa_, similar to PhaJ_Ac_, supplies C_4_-C_6_-chain-length precursors for PHA polymerization, whereas PhaJ4_Pa_ supplies medium-chain-length (C_6_-C_10_) precursors^12^. In *Ralstonia eutropha*, as many as 16 orthologs of R-ECHs were detected in the genome, among which three R-ECHs (PhaJ4a_Re_ to PhaJ4c_Re_) sharing high homologies with PhaJ4_Pa_ were characterized^6^, indicating the R-ECH redundancy that is involved in PHA biosynthesis.

As the hydration reaction catalyzed by R-ECHs is a reversible process^1^, R-ECHs may be involved in PHA degradation as well as in PHA biosynthesis. In *R. eutropha* H16, the *in vitro* degradation products of the native PHB (nPHB) granule contain (*S*)-3-hydroxybutyryl-CoA [(*S*)-3HB-CoA] and crotonyl-CoA, while in recombinant *Escherichia coli*, the corresponding products are (*R*)-3HB-CoA and crotonyl-CoA^13^. Therefore, putative R-ECH and S-ECH may mingle with or locate on these PHA granules. The putative R-ECH catalyzes the dehydration of (*R*)-3HB-CoA to crotonyl-CoA, and the latter compound is further converted to (*S*)-3HB-CoA by the putative S-ECH. As crotonyl-CoA and (*S*)-3HB-CoA are intermediates of the β-oxidation cycle, the PHB degradation is putatively connected by crotonyl-CoA to β-oxidation in *R. eutropha*^13^, although the putative R-ECH^6^,^14^ was not identified yet.

Comparing to the R-ECH research in bacteria, the characterization of R-ECH in archaea was barely known until recently. Previously, six PHA-granule-associated proteins (PGAPs)^15^,^16^ were identified in the haloarchaeon *Haloferax mediterranei*, a model strain for PHA metabolism research in archaea^17^,^18^,^19^. The six PGAPs include putative R-ECH (HFX_5217), phasin PhaP^15^, regulatory protein PhaR^20^, PHA synthase subunits PhaE and PhaC^21^, and PHA depolymerase PhaZh1^16^. Among these PGAPs, only the function of HFX_5217 was not characterized yet in *H. mediterranei*. Interestingly, the genes of the former five PGAPs form a gene cluster (*HFX_5217*-*phaR*-*phaP*-*phaE*-*phaC*) in the genome^15^. The organization of this gene cluster suggests that HFX_5217 may function in the PHA metabolism in *H. mediterranei*.

Recently, bioinformatics analysis demonstrated that the gene candidates of the β-oxidation pathway are widely present in haloarchaea and in a few other archaea^22^. However, as many haloarchaea seem ineffective in utilizing the environmental fatty acids, the physiological function of the β-oxidation cycle in haloarchaea remains unclear. In this study, through gene knockout, gene complementation and enzyme assays, we showed that PHA mobilization in *H. mediterranei* could be linked to the β-oxidation cycle by an R-ECH (HFX_5217, named PhaJ1 in this study). We have further explored the distribution of key genes that are involved in PHA metabolism and β-oxidation in all of the sequenced haloarchaea species that also encode PhaJ. The results implied that haloarchaea may generally use the PhaJ-linked PHA mobilization and β-oxidation as a flexible adaptation to the changeable carbon sources in high-salt environments.

## Results

### Analysis of the R-ECH homologous proteins in *H. mediterranei*

**Figure 1:**
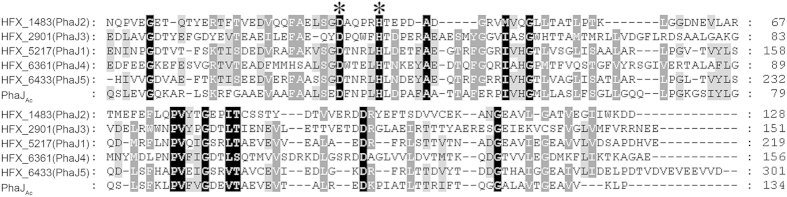
Alignment of the amino acid sequences of (*R*)-specific enoyl-CoA hydratase (R-ECH) homologous proteins in *H. mediterranei*. HFX_1483 (GenBank accession number YP_006349178), HFX_2901 (YP_006350558), HFX_5217 (YP_006351037), HFX_6361 (YP_006351469), and HFX_6433 (YP_006351539) are the five putative R-ECHs in *H. mediterranei*. PhaJ_Ac_ (BAA21816) is the R-ECH in *Aeromonas caviae*. The putative active sites are shown with asterisks. The numbers on the right are the positions of the amino acids of the respective proteins. Identical and similar residues are shaded in black and gray, respectively.

To elucidate the possible relationship between PHA metabolism and the β-oxidation cycle in *H. mediterranei*, a bioinformatics analysis of R-ECHs in the genome of *H. mediterranei*^18^ was performed, and five genes (*HFX_1483*, *2901*, *5217*, *6361*, and *6433*) that encode the putative R-ECH homologous proteins (Fig. 1) were detected. All five proteins contain a MaoC-like domain (pfam01575), similar to PhaJ_Ac_, which is involved in PHA biosynthesis for supplying (*R*)-3HB-CoA from fatty acid β-oxidation^4^,^11^. HFX_6433 (301 amino acids) and HFX_5217 (219 amino acids) show 45% (coverage 45%) and 41% (coverage 57%) identities to PhaJ_Ac_ (134 amino acids), respectively, while the other three proteins share relatively low (<30%) identities with the PhaJ_Ac_. According to the structure of PhaJ_Ac_^11^, putative active sites (aspartate and histidine) were found in all of the five proteins (Fig. 1). As only the HFX_5217 of these five R-ECHs is associated with the PHA granules^15^, we designated HFX_5217 the PhaJ1 of *H. mediterranei*. The other four proteins (HFX_1483, 2901, 6361, and 6433) were named PhaJ2 to PhaJ5, respectively (Fig. 1). This bioinformatics analysis provides the candidate PhaJs that may be involved in PHA metabolism in *H. mediterranei*.

### Effect of PhaJs on PHA synthesis in *H. mediterranei*

**Figure 2:**
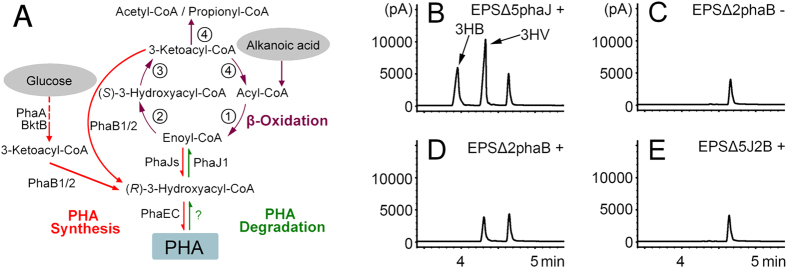
PHA biosynthesis in *H. mediterranei* from valeric acid. (**A**) Proposed PHA metabolism pathway in *H. mediterranei* from glucose^49^ and alkanoic acid. PhaA/BktB^50^, β-ketothiolases; PhaB1/2^27^, acetoacetyl-CoA reductases; PhaEC^21^, PHA synthase; PhaJ, (*R*)-specific enoyl-CoA hydratase. The dashed line indicates involvement of multiple enzymes. The question mark (?) indicates that the enzyme that catalyzes the reaction is unknown. The predicted enzymes of ① acyl-CoA dehydrogenase, ② (*S*)-enoyl-CoA hydratase, ③ 3-hydroxyacyl-CoA dehydrogenase, and ④ 3-ketoacyl-CoA thiolase that were involved in β-oxidation pathway in *H. mediterranei* are listed in Supplementary Information Table S3. (**B–E**) GC analysis of the effect of PhaBs and PhaJs on PHA accumulation in *H. mediterranei* with or without valeric acid in the medium. Mutant strains were *H. mediterranei* EPSΔ5phaJ (**B**), EPSΔ2phaB (**C**,**D**), and EPSΔ5J2B (**E**). Different monomers (3-hydroxybutyrate, 3HB and 3-hydroxyvalerate, 3HV) are shown with arrows. The peak at 4.7 min represent methyl benzoate, which is used as an internal standard for quantitative calculation. ‘−’ and ‘+’ indicate without and with valeric acid added to the medium, respectively. pA indicates picoampere.

In haloarchaea, the degradation of long-chain fatty acids is currently unknown, and β-oxidation might be limited to short-chain fatty acids^23^. Interestingly, short-chain fatty acids, such as butyric acid, valeric acid (pentanoic acid) and hexanoic acid (caproic acid), can be used as the sole carbon source for cultivating *H. mediterranei*. As observed in bacteria^24^, *H. mediterranei* can also accumulate PHBV with a high ratio of the 3-hydroxyvalerate (3HV) unit when valeric acid was added to the medium^25^,^26^. This high fraction of 3HV in PHBV may come from the contribution of either PhaJ(s)^4^,^5^,^6^,^12^ or PhaBs^27^, as they can supply (*R*)-3HA-CoA (i.e., (*R*)-3HV-CoA from valeric acid) from different β-oxidation intermediates, enoyl-CoA (i.e., 2-pentenoyl-CoA from valeric acid) or 3-ketoacyl-CoA (i.e., 3-ketovaleric-CoA from valeric acid), respectively (Fig. 2A).

To identify the PhaJ(s) that might be involved in the PHBV biosynthesis in *H. mediterranei*, we deleted the five *phaJs* in *H. mediterranei* EPS individually (Supplementary Information Table S1). GC analysis (Table 1) revealed that the single *phaJ* mutant strains accumulated PHBV with the similar ratio of 3HV as the control strain. This result indicates that the deletion of single *phaJ* in *H. mediterranei* has no significant effect on PHBV accumulation. In considering the redundancy of PhaJs^6^,^12^, we deleted all five *phaJs* in *H. mediterranei* EPS. However, the 3HV ratio of the PHBV accumulated in the mutant strain *H. mediterranei* EPSΔ5phaJ also did not decrease (Table 1 and Fig. 2B), indicating that the PhaJ-route is unlikely the main pathway for supplying 3HV-CoA from valeric acid for PHBV biosynthesis in *H. mediterranei*.

To evaluate the contribution of PhaB1/PhaB2^27^ in converting another intermediate of valeric acid β-oxidation, 3-ketovaleryl-CoA, into (*R*)-3HV-CoA for PHBV biosynthesis (Fig. 2A), we then deleted the two *phaBs* in *H. mediterranei* EPS. The double-*phaBs* mutant strain *H. mediterranei* EPSΔ2phaB lost the ability to accumulate PHA when the cells were grown in PAC medium (see Methods) as previously reported^27^ (Fig. 2C). But interestingly, a small amount of PHV (0.12 ± 0.07 g L^−1^) was accumulated in *H. mediterranei* EPSΔ2phaB when grown in PAC medium with valeric acid added (Fig. 2D). We further deleted the two *phaBs* in *H. mediterranei* EPSΔ5phaJ, resulting in the mutant strain *H. mediterranei* EPSΔ5J2B. Notably, the *H. mediterranei* EPSΔ5J2B cannot accumulate either PHBV or PHV in the cells when grown in PAC medium even when valeric acid is added (Fig. 2E). These results indicate that the metabolic flux of (*R*)-3HV-CoA from valeric acid for PHV biosynthesis in *H. mediterranei* EPSΔ2phaB comes from the contribution of PhaJs. However, comparing the large amount of PHBV accumulated in *H. mediterranei* EPSΔ5phaJ (1.03 ± 0.07 g L^−1^, Fig. 2B) and little amount of PHV in *H. mediterranei* EPSΔ2phaB (0.12 ± 0.07 g L^−1^, Fig. 2D), it is clear that the PhaB-route had much more contribution than the PhaJ-route to the metabolic flux of 3HV-CoA from valeric acid.

**Figure 3:**
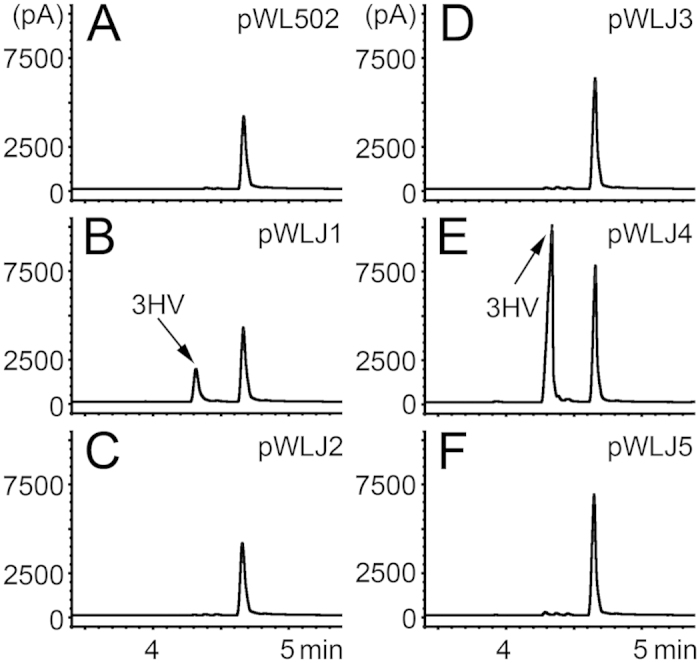
Effect of *phaJs* complementation on PHV accumulation in the mutant *H. mediterranei* EPSΔ5J2B. The mutant strain harboring plasmid pWL502 (empty plasmid, (**A**)), pWLJ1 (**B**), pWLJ2 (**C**), pWLJ3 (**D**), pWLJ4 (**E**), and pWLJ5 (**F**), individually, were grown in PAC medium with valeric acid added. The monomer (3-hydroxyvalerate, 3HV) is shown with an arrow. The peak at 4.7 min represents methyl benzoate, which is used as an internal standard for quantitative calculation. pA indicates picoampere.

To distinguish which one of these PhaJs is involved in the PHA biosynthesis, the *H. mediterranei* EPSΔ5J2B strain was individually complemented with these *phaJs* (Fig. 3). Interestingly, only the *phaJ1* (Fig. 3B) and *phaJ4* (Fig. 3E) complementation strains recovered PHV accumulation in the cells, and more PHV accumulated in the *phaJ4* (0.28  ± 0.01 g L^−1^, Fig. 3E) complementation strain than in the *phaJ1* (0.07 ± 0.02 g L^−1^, Fig. 3B) complementation strain.

These results demonstrate that certain PhaJs (mainly PhaJ4) are involved in but had little contribution to the metabolic flux of (*R*)-3HA-CoA for PHA synthesis in *H. mediterranei*.

### Effect of PhaJs on PHA mobilization in *H. mediterranei*

As PhaJs contribute little to PHA synthesis and the reaction that is catalyzed by R-ECH is a reversible process, PhaJs may be significantly involved in PHA mobilization in *H. mediterranei*. We investigated the effect of PhaJs on PHA mobilization in *H. mediterranei* using the *phaJs* sole- and multiple-deletion mutant strains. GC analysis (Table 2) showed that the strain *H. mediterranei* EPSΔphaJ1 significantly decreased PHA mobilization compared to the control strain *H. mediterranei* EPS. However, other *phaJs* sole-deletion mutant strains had no effect on decreasing PHA mobilization in *H. mediterranei* (Table 2). Thus, even the *phaJs* multiple deletion mutant strain *H. mediterranei* EPSΔ5phaJ did not further decrease the PHA mobilization compared to the mutant strain *H. mediterranei* EPSΔphaJ1 (Table 2). This result indicates that only PhaJ1, among the five PhaJs, is involved in PHA mobilization in *H. mediterranei*.

**Figure 4:**
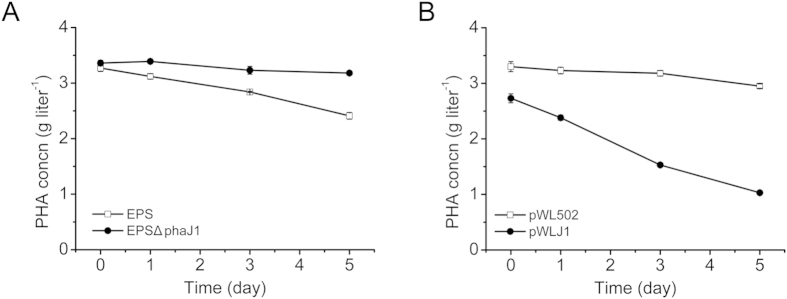
Effect of *phaJ1* deletion (**A**) and complementation (**B**) on PHA mobilization in *H. mediterranei*. EPS, *H. mediterranei* EPS; EPSΔphaJ1, *H. mediterranei* EPSΔphaJ1. pWL502, *H. mediterranei* EPSΔphaJ1 harboring empty plasmid; and pWLJ1, *H. mediterranei* EPSΔphaJ1 harboring expression plasmid of *phaJ1* with its promoter region.

To confirm the function of PhaJ1 on PHA mobilization, *phaJ1* gene complementation was performed in *H. mediterranei* EPSΔphaJ1. GC analysis demonstrated that the mutant strain *H. mediterranei* EPSΔphaJ1 utilized much less amount of accumulated PHA than that of the wild-type *H. mediterranei* EPS (Fig. 4A). In contrast, compared to the strain *H. mediterranei* EPSΔphaJ (pWL502) harboring empty plasmid, the *phaJ1* complementation strain *H. mediterranei* EPSΔphaJ1 (pWLJ1) harboring the PhaJ1-expression plasmid significantly increased PHA degradation (62.3% *vs* 10.6% degradation at 5^th^ day, Fig. 4B).

These results further demonstrate that PhaJ1 is involved in PHA mobilization in *H. mediterranei* and would act as an important enzyme of PHA degradation pathway.

### Expression, purification, and functional analysis of PhaJ1

**Figure 5:**
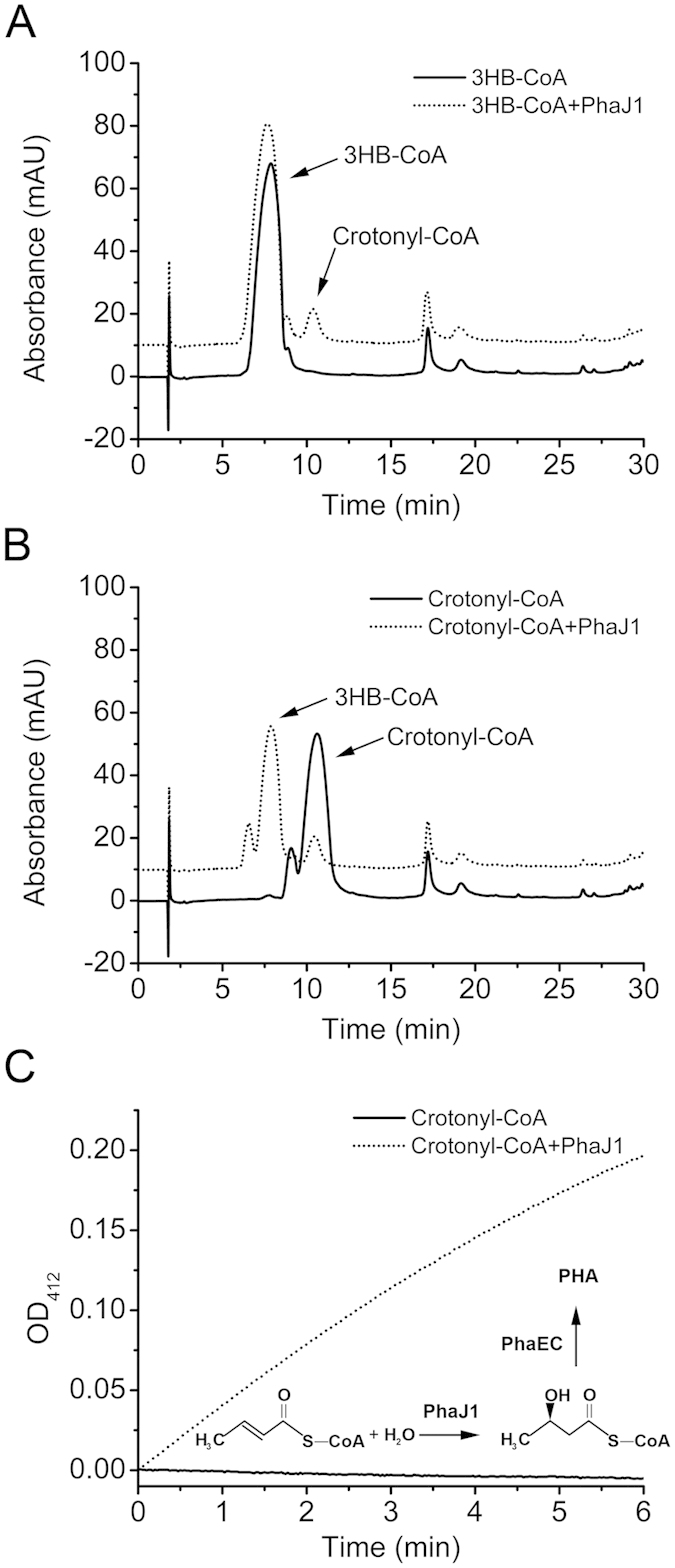
Enzyme assay of PhaJ1 with the substrates of 3-hydroxybutyryl-CoA (3HB-CoA) and crotonyl-CoA. HPLC analysis of the products of dehydration and hydration of 3HB-CoA (**A**) and crotonyl-CoA (**B**), respectively, as catalyzed by PhaJ1. (**C**) Evaluation of the stereospecificity of PhaJ1. The hydration of crotonyl-CoA as catalyzed by PhaJ1 coupled with crude PHA synthase located on nPHA granules that were isolated from *H. mediterranei* EPSΔphaJ1 was spectrophotometrically monitored at 412 nm.

To determine the biochemical activity of PhaJ1, we expressed and purified PhaJ1 with N-terminal His-tags (PhaJ1-His_6_) as described in Methods. The products of 3HB-CoA dehydration and crotonyl-CoA hydration as catalyzed by PhaJ1 were identified via HPLC assay (Fig. 5A,B). In the 3HB-CoA dehydration reaction (Fig. 5A), minor 3HB-CoA was converted into crotonyl-CoA by PhaJ1. Correspondingly, in the crotonyl-CoA hydration reaction (Fig. 5B), major crotonyl-CoA was converted into 3HB-CoA by PhaJ1. This result demonstrates that the hydration and dehydration between crotonyl-CoA and 3HB-CoA as catalyzed by PhaJ1 is a reversible reaction and that the hydration process is the main reaction *in vitro*.

The stereospecificity of PhaJ1 was evaluated by crotonyl-CoA hydration coupled with crude PHA synthase^4^,^6^ of *H. mediterranei* (PhaEC_Hme_), which catalyzed (*R*)-3HA-CoA polymerization^21^. Linked with the (*R*)-3HB-CoA polymerization that is catalyzed by PhaEC_Hme_, the released CoA-SH was monitored at 412 nm (TNB-CoA) in the presence of DTNB^21^. The TNB-CoA was clearly observed when purified PhaJ1 was added to the reaction mixture containing crotonyl-CoA, crude PhaEC_Hme_, and DTNB, while the control (without PhaJ1 added) had no change in absorbance at 412 nm in the same reaction condition (Fig. 5C). This result demonstrates that the hydration of enoyl-CoA to 3HA-CoA as catalyzed by PhaJ1 has (*R*)-specificity.

Thus, through converting (*R*)-3HA-CoA, the common product of PHA degradation, to enoyl-CoA, the intermediate of the β-oxidation cycle, PhaJ1 could link PHA mobilization to β-oxidation cycle, and finally generate acetyl-CoA or propionyl-CoA for central metabolism in *H. mediterranei* (Fig. 2A).

### Involvement of the β-oxidation pathway in PHA mobilization

The above results implied that the PHA degradation product, (*R*)-3-HA-CoA, could enter the β-oxidation pathway as mediated by PhaJ1. To investigate whether β-oxidation pathway is indeed involved in PHA re-utilization in *H. mediterranei*, we performed an inhibition assay as well as a qRT-PCR assay of the β-oxidation pathway under the carbon limited (PHA mobilization) condition.

**Figure 6:**
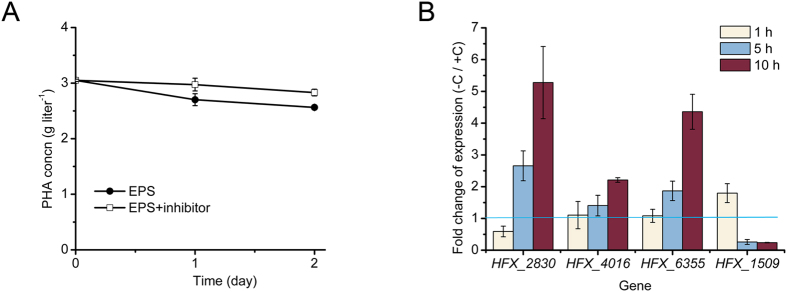
The involvement of β-oxidation pathway in PHA mobilization in *H. mediterranei*. (**A**) The inhibitors of β-oxidation enzyme inhibited PHA mobilization in *H. mediterranei*. EPS, *H. mediterranei* EPS; Inhibitor, acrylic acid (11 mM) plus thioglycolic acid (1.2 mM). (**B**) qRT-PCR assay for analysis of the fold change of gene expression when *H. mediterranei* EPS cultured in fresh PAC medium without (−C) *vs* with (+C) glucose. Genes *HFX_1509*, *HFX_2830*, *HFX_4016*, and *HFX_6355* encoding the 3-hydroxyacyl-CoA dehydrogenase in β-oxidation cycle were chosen. The fold change of gene expression at 1 h, 5 h, and 10 h was analyzed. The fold change of expression of each gene was calculated by normalization to the expression of inner control 7S RNA.

To inhibit the β-oxidation pathway, the inhibitors of its key enzymes 3-ketoacyl-CoA thiolase and acyl-CoA dehydrogenase, i.e. acrylic acid^28^ and thioglycolic acid^29^, were supplied together in the PHA mobilization assay. It is clearly indicated that after addition of these inhibitors, the PHA mobilization immediately slowed down (from the data of the first two days after inhibitor addition) in *H. mediterranei* as expected (Fig. 6A).

Furthermore, we have also analyzed the gene expression regulation of the β-oxidation pathway in PHA mobilization. The expression of four genes (*HFX_1509*, *HFX_2830*, *HFX_4016*, and *HFX_6355*, Supplementary Information Table S3) that encode the predicted key enzyme, 3-hydroxyacyl-CoA dehydrogenase, in β-oxidation cycle were investigated when the PHA-accumulated cells were cultured under the carbon-limited *vs* carbon-enriched conditions (Fig. 6B). After cells were transferred in PD medium (PAC medium without glucose, the condition for PHA mobilization), the expression of gene *HFX_2830*, *HFX_4016*, and *HFX_6355* was up-regulated clearly and enhanced gradually within the observed 10 hours, while the expression of gene *HFX_1509* was up-regulated in the first hour but decreased later (Fig. 6B). These results indicated that the first three genes (*HFX_2830*, *HFX_4016*, and *HFX_6355*) may encode the major 3-hydroxyacyl-CoA dehydrogenase functioned in β-oxidation pathway in PHA mobilization, while the *HFX_1509* responded rapidly but may only play the role at the beginning of PHA mobilization. Notably, the up-regulation of the key genes of β-oxidation pathway in PHA mobilization further implied the involvement of β-oxidation pathway in PHA mobilization in *H. mediterranei*.

### Distribution of phaJ, phaC and the β-oxidation cycle in haloarchaea

**Figure 7:**
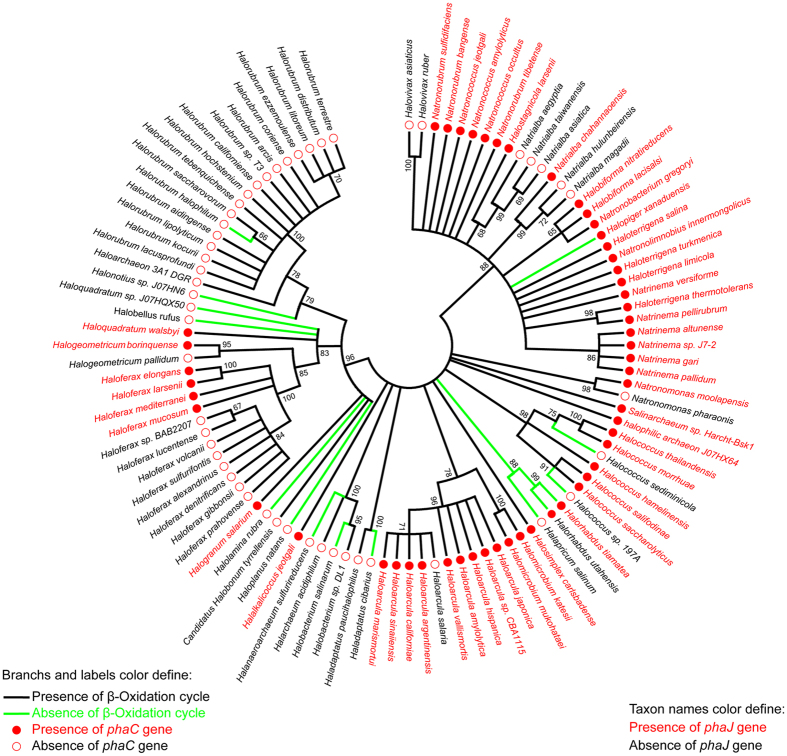
Maximum-likelihood phylogenetic tree of class *Halobacteria* constructed on the alignment of amino acid sequences of RNA polymerase subunit B′ (RpoB′). Bootstrap values >50%, as calculated from 1,000 replications, are shown at the tree branches. The tree shows, distinguished by different colors, the distribution of the genes encoding PhaJ [(*R*)-specific enoyl-CoA hydratase], PhaC (PHA synthase) and the characteristic enzymes that are involved in β-oxidation cycle (acyl-CoA dehydrogenase, enoyl-CoA hydratase, 3-hydroxyacyl-CoA dehydrogenase, and 3-ketoacyl-CoA thiolase) in the class *Halobacteria*.

To address whether the PhaJ-linked PHA degradation to β-oxidation cycle is an individual case in *H. mediterranei* or may be a general case in the class *Halobacteria*, we analyzed the distribution of *phaJ*, *phaC* and the genes for the β-oxidation cycle in the genomes of 103 sequenced haloarchaea species. The genes *phaJ* and *phaC* were found in the genomes of 52 species (50%) and 53 species (51%) of haloarchaea (Fig. 7), respectively. Only one species (*Halorhabdus utahensis*) containing *phaC* in the genome did not possess *phaJ*; 100% of the species possessing *phaJ* also contained *phaC* in the genome. The full set of genes for the four key enzymes of the β-oxidation cycle (acyl-CoA dehydrogenase, enoyl-CoA hydratase, 3-hydroxyacyl-CoA dehydrogenase and 3-ketoacyl-CoA thiolase) were found in the genomes of 88 species (85%) (Fig. 7). In the 52 species that contained *phaJ* in the genome, only two species (*Halopiger xanaduensis* and *Halorhabdus tiamatea*) do not have the full set of genes for the characteristic enzymes of the β-oxidation cycle. Therefore, 96% of species containing *phaJ* (100% containing *phaC*) also possess a full set of genes for the β-oxidation cycle. This result suggests that PhaJ linking PHA degradation to the β-oxidation cycle would be a general case in the class *Halobacteria*.

## Discussion

PHA accumulated in cells as carbon and energy sources can be mobilized under carbon starvation conditions^30^. Mobilizing the accumulated PHA in cells is an important mechanism for haloarchaea thriving in hypersaline environments with changeable carbon sources. However, the PHA degradation pathways in haloarchaea are still not clear. Recently, we found a novel PHA depolymerase PhaZh1 in *H. mediterranei*^16^. However, although PhaZh1 showed high activity in degradation of the PHB(V) into 3-hydroxybutyrate and was most likely the key enzyme in nPHA granule hydrolysis *in vitro*, the knockout of *phaZh1* had no significant effect on the intracellular PHA mobilization, implying the existence of alternative PHA mobilization pathway(s) that function more effectively within the cells of *H. mediterranei*^16^. In this study we showed that an R-ECH (PhaJ) played as a major enzyme in mediating PHA mobilization in *H. mediterranei*.

In bacteria, a recent study has shown that the PHB degradation is associated with the β-oxidation cycle via crotonyl-CoA^13^, but the enzyme (likely an R-ECH) catalyzing (*R*)-3HB-CoA to crotonyl-CoA had not been identified. Interestingly in *H. mediterranei*, a putative R-ECH (HFX_5217, named PhaJ1 in this study) was recently found on PHA granules. The gene of HFX_5217 was within the *pha*-cluster (*HFX_5217*-*phaR*-*phaP*-*phaE*-*phaC*) in the genome, which indicates that its function may involve in PHA metabolism^15^. Meanwhile, the other four putative R-ECHs (PhaJ2 to PhaJ5) genes are dispersed in the genome of *H. mediterranei*. Certain PhaJs (i.e., PhaJ4 and PhaJ1) in *H. mediterranei* were indeed involved in PHA biosynthesis (Fig. 3B,E), as observed in bacteria^4^,^6^,^31^,^32^, but showed a minor contribution in the formation of (*R*)-3HV-CoA through valeric acid β-oxidation, as compared to the pathway directed by PhaBs (Fig. 2). This may be probably because PhaJs have a lower affinity toward 2-pentenoyl-CoA than that of PhaBs toward 3-ketovaleryl-CoA, and the reduction reaction that was catalyzed by PhaBs consumed NAD(P)H^33^, allowing the process to more easily continue.

As mentioned above, R-ECHs reversibly catalyze the hydration reaction^1^. Thus, as PHA is usually degraded to 3HA-CoA *in vivo* in the presence of CoA^13^,^34^, the linkage between PHA mobilization and β-oxidation mediated by PhaJs would be feasible in *H. mediterranei*. Among all of the single- or multiple-*phaJs* deletion mutants, *H. mediterranei* EPSΔphaJ1 and EPSΔ5phaJ significantly decreased the PHA mobilization compared to wild-type and other *phaJ* mutants (Table 2). This may be due to PhaJ1 having a higher catalytic efficiency than other PhaJs. Moreover, only PhaJ1 is located on PHA granules^15^ and thus has more opportunity than other PhaJs to catalyze the substrate (*R*)-3HA-CoA to enoyl-CoA. Furthermore, the PHA mobilization in the *phaJ1* complementation strain was significantly accelerated, demonstrating that PhaJ1 indeed plays a major role in PHA mobilization in *H. mediterranei* (Fig. 4).

Interestingly, in the *in vitro* assay, only a few crotonyl-CoA are produced in the 3HB-CoA dehydration, while nine-tenths of crotonyl-CoA were converted into (*R*)-3HB-CoA by PhaJ1 in the hydration reaction, indicating that PhaJ1 prefers hydration to dehydration *in vitro* (Fig. 5). However, the PhaJ1 *in vivo* was demonstrated as an important enzyme in PHA mobilization (Table 2 and Fig. 4), which catalyzed the dehydration reaction. This may be due to that the enzyme assay *in vitro* does not reflecting the real conditions *in vivo*. As H_2_O is one product of dehydration, thus dehydration reactions in aqueous solutions are difficult to carry out *in vitro*. However, crotonyl-CoA that is produced during dehydration by PhaJ1 can be further metabolized via the β-oxidation pathway in the cells; therefore, the dehydration reaction can be continued and more efficient *in vivo* as observed.

As in the medium without fatty acid and other carbon resources, the up-regulation of genes involved in β-oxidation pathway (Fig. 6B) implied that these genes could be induced by inner carbons generated from the PHA mobilization. The 3HA-CoA generated from PHA degradation could be converted to enoyl-CoA by PhaJ1 (Fig. 5A) and then enters into the β-oxidation pathway. This was further confirmed that the PHA degradation can be weakened by the β-oxidation enzyme inhibitors (Fig. 6A). Furthermore, the genome analysis of the sequenced haloarchaea species implied that PHA-accumulating haloarchaeal strains may mobilize the storage compound during carbon starvation through the β-oxidation cycle as mediated by PhaJ1 (Figs 7 and 2A). The final products of acetyl-CoA and propionyl-CoA could be assimilated efficiently via methylaspartate cycle or glyoxylate cycle^35^,^36^ and the propionyl-CoA carboxylation pathway^37^ in many haloarchaea. Integration of the PHA mobilization to either general β-oxidation cycle or the following specific pathways may reflect the evolutionary adaptation of haloarchaea to a high-salt environment.

In summary, unlike (*R*)-specific enoyl-CoA hydratase PhaJs in bacteria, which are mainly involved in PHA synthesis^4^,^6^,^31^,^32^, PhaJs in *H. mediterranei* have little contribution to PHA accumulation. In contrast, PhaJ1 mediated the linkage of PHA degradation to the β-oxidation pathway by catalyzing the dehydration of (*R*)-3HA-CoA to enoyl-CoA in *H. mediterranei* (Fig. 2A), and this case may be general in the class *Halobacteria* (Fig. 7). Following different pathways, simultaneous PHA synthesis and degradation^38^,^39^ may occur without a loss of energy in haloarchaea. Therefore, this PHA metabolism mechanism in haloarchaea may help PHA-accumulating species to adapt to extreme environments, especially during carbon starvation.

## Methods

### Strains, culture conditions and plasmids

The strains and plasmids that were used in this study are listed in Supplementary Information Table S1. *E. coli* strains were grown at 37 °C in Lysogeny Broth (LB) medium. *E. coli* JM109^40^ and JM110^41^ were used as host strains for general gene cloning and for the construction of unmethylated plasmids, respectively. *H. mediterranei* EPS^17^ (a gene cluster involved in exopolysaccharide (EPS) synthesis was deleted, thus producing more PHA than the wild-type *H. mediterranei*) and their recombinant strains harboring plasmids were cultivated at 37 °C in AS-168 medium^42^ and AS-168SY medium^19^, respectively, for 48 h as seed culture. Then, the seed culture was transferred [1:25 inoculation, (vol/vol)] into PAC medium [per liter, NaCl 110 g, MgSO_4_⋅7H_2_O 29.52 g, MgCl_2_⋅6H_2_O 20.51 g, KCl 5 g, CaCl_2_ 1 g, NH_4_Cl 2 g, KH_2_PO_4_ 0.0375 g, glucose 10 g, 1,4-piperazinediethanesulfonic acid (PIPES) 15 g, ammonium ferric citrate 0.008 g, 1 ml of trace element solution SL-6^17^] and further transferred into PD medium (PAC medium without glucose) for PHA accumulation and degradation research as previously described^16^. *Haloferax volcanii* strains were grown at 45 °C in Hv-YPC medium^43^. Ampicillin (100 μg ml^−1^) and thymidine (40 μg ml^−1^) were added to the LB and Hv-YPC media, respectively, when necessary. Uracil (50 μg ml^−1^) was supplied to the medium when the *H. mediterranei* EPS^17^ or its mutants were cultivated.

### Gene deletion and complementation

The primers that were used for gene amplification in this study are listed in Supplementary Information Table S2. The plasmids (based on the *pyrF* selection, Supplementary Information Table S1) pHFX^19^ and pWL502^15^ were used for gene deletion and expression, respectively. DNA manipulations were performed according to standard methods. The genes that were cloned by PCR amplification were confirmed by DNA sequencing. After being shuttled into and isolated from *E. coli* JM110, the plasmid was introduced into *H. mediterranei* EPS or its mutants. The recombinant *H. mediterranei* EPS or its mutant strain was screened and identified as previously described^19^.

### PHA accumulation and degradation assay

The PHA accumulation and degradation analysis was performed as previously described^16^. Briefly, the seed culture in AS-168 or AS-168SY medium was transferred [1:25 inoculation, (vol/vol)] into PAC medium and grown for 3 days for the PHA accumulation assay. For the PHA degradation analysis, the strains that accumulated PHA were resuspended and incubated in PD medium for 5 days.

Valeric acid was used as the carbon source in the medium to improve the 3HV content of the PHA that accumulated in *H. mediterranei*. For the PHA accumulation assay, valeric acid (final concentration 15 mM, pH 7.0) was added when the strains were grown in PAC medium for 2 days, and the strains were collected after another 2 days (total cultivation-time, 4 days).

Acrylic acid^28^ (final concentration 11 mM, pH 7.0) and thioglycolic acid^29^ (final concentration 1.2 mM, pH 7.0), the inhibitors of 3-ketoacyl-CoA thiolase and acyl-CoA dehydrogenase, respectively, were used together in PHA degradation assay to investigate the involvement of β-oxidation in PHA mobilization.

The PHA concentration and content were analyzed by gas chromatography (GC) (GC-6820, Agilent, USA) assay as previously described^42^.

### Expression and purification of PhaJ1-His_6_ in *H. volcanii*

The expression plasmid pTA05 (Supplementary Information Table S1) was used for PhaJ1 expression and purification in *H. volcanii* as previously described^16^. Briefly, the plasmid pTA05-phaJ1 isolated from *E. coli* JM109 was transformed into *H. volcanii* H1424^43^. After harvest (at 4 °C), the recombinant strain was ultrasonicated. PhaJ1-His_6_ in the supernatant was purified by a Ni-agarose column at 4 °C. The matrix-assisted laser desorption ionization-tandem time of flight mass spectrometry as described by Shevchenko *et al*.^44^ was used to identify the purified protein.

The protein concentration was measured at 562 nm using a spectrophotometer (DU800; Beckman Coulter, USA) and the bicinchoninic acid method^45^.

### Enzyme assay

The enoyl-CoA hydratase and dehydratase activity of PhaJ1 was assayed by the hydration of crotonyl-CoA (Sigma)^4^,^46^ and the dehydration of 3HB-CoA (racemate, Sigma)^46^, respectively. Briefly, a total volume of 500 μl of reaction mixture contains 2 M KCl, 100 mM Tris-HCl (pH 7.5), 5 mM MgCl_2_, 0.25 mM crotonyl-CoA or 3HB-CoA, and 20 μg of purified PhaJ1-His_6_. After adding the protein into the mixture, the reaction was started at 45 °C. The reaction was stopped and acidified with 0.1 N HCl after 30 min.

After being filtered through a 0.22 μm syringe filter, the reaction products were analyzed by high-performance liquid chromatography (HPLC) (HPLC-1220, Agilent, USA) as previously described^13^ with a slight change. The conditions for the HPLC analysis were as follows: Eclipse XDB-C_18_ column (5 μm, 4.6 mm by 150 mm, Agilent Technologies); column temperature, 40 °C; injection volume, 20 μl; and detection wavelength, 254 nm. For the separation of the crotonyl-CoA and 3HB-CoA, the column was flushed at a flow rate of 0.8 ml min^−1^ by a linear gradient from 5% (vol/vol) acetonitrile (CH_3_CN) in 50 mM ammonium acetate (pH 4.7) to 50% CH_3_CN within 25 min, followed by up to 80% CH_3_CN within 5 min (total run time, 30 min), followed by a decrease to 5% CH_3_CN within 8 min (total run time, 38 min) and an isocratic flow of 5% CH_3_CN for 4 min (total run time, 42 min).

The hydration of crotonyl-CoA as catalyzed by PhaJ1 coupled with crude PHA synthase on nPHA granules^15^,^21^ that were isolated from *H. mediterranei* EPSΔphaJ1 was monitored to evaluate the stereospecificity of enoyl-CoA hydratase as previously described^4^,^6^. Briefly, the nPHA granules were isolated via sucrose density gradient centrifugation as previously described^15^. The reaction mixture (500 μl) contained nPHA granules [final optical density at 650 nm (OD_650_), ~0.16], 2 M KCl, 100 mM Tris-HCl (pH 7.5), 5 mM MgCl_2_, 0.25 mM crotonyl-CoA, 0.2 mM 5,5’-dithiobis-(2-nitrobenzoic acid) (DTNB), and 40 μg of purified PhaJ1-His_6_. After the protein was added, the TNB-CoA that was produced in the reaction solution was monitored spectrophotometrically (DU800; Beckman Coulter, USA) at 412 nm^21^.

### RNA extraction and quantitative reverse transcription-PCR (qRT-PCR)

The PHA-rich cells of *H. mediterranei* EPS were harvested and cultured in fresh PAC medium with or without glucose, and the total RNA of *H. mediterranei* EPS cells at 1 h, 5 h, and 10 h was extracted with TRIzol reagent (Invitrogen, USA) as previously described^47^. TURBO DNA-free™ Kit (Thermo Fisher Scientific, USA) was used for removing DNA contamination. The cDNA was synthesized by reverse transcription with random hexamer primers from 1 μg of DNA-free total RNA using the Moloney Murine Leukemia Virus Reverse Transcriptase (M MLV-RT) (Promega, USA). The fold change of gene expression was analyzed by ViiA™ 7 Real-Time PCR System (ABI, USA), using the levels of 7S RNA (always in constitutive expression) as an endogenous control to normalize the data resulting from each sample. The primers were listed in Supplementary Information Table S2.

### Protein sequence alignment and phylogenetic analysis

The homology of protein sequences was analyzed by the GeneDoc program (http://www.nrbsc.org/old/gfx/genedoc/). The phylogenetic tree was constructed by the MEGA6 program using a maximum likelihood algorithm with 1,000 bootstrap of replications^48^. Query sequences were acquired from the National Center for Biotechnology Information (NCBI) database, and the BLASTp program was used for protein BLAST (http://www.ncbi.nlm.nih.gov/BLAST/). The GenBank accession numbers of RNA polymerase subunit B′ (RpoB′) are listed in Supplementary Information Table S4.

## Additional Information

**How to cite this article**: Liu, G. *et al*. Enoyl-CoA hydratase mediates polyhydroxyalkanoate mobilization in *Haloferax mediterranei*. *Sci. Rep*. **6**, 24015; doi: 10.1038/srep24015 (2016).

## Supplementary Material

Supplementary Information

## Figures and Tables

**Table 1:** Effect of PhaJs on PHA synthesis in *H. mediterranei*.

Strain	PHBV content (% wt/wt)	3HV fraction (mol%)	Cell dry wt (g L^−1^)
EPS	58.80 ± 2.58	29.72 ± 1.24	6.35 ± 0.16
EPSΔphaJ1	58.34 ± 1.99	27.97 ± 0.44	6.50 ± 0.13
EPSΔphaJ2	61.09 ± 1.42	30.86 ± 0.37	6.03 ± 0.05
EPSΔphaJ3	60.14 ± 1.31	30.25 ± 0.81	6.35 ± 0.03
EPSΔphaJ4	58.21 ± 3.90	30.67 ± 2.08	6.21 ± 0.14
EPSΔphaJ5	54.31 ± 4.60	34.38 ± 4.06	5.82 ± 0.46
EPSΔ5phaJ	61.98 ± 2.66	32.18 ± 1.28	6.45 ± 0.19

**Table 2:** Effect of PhaJs on PHA mobilization in *H. mediterranei*.

Strain	PHA concn (g L^−1^)		PHA mobilization
	Day 0	Day 5	(%)
EPS	3.42 ± 0.09	2.54 ± 0.04	25.71
EPSΔphaJ1	3.14 ± 0.05	2.96 ± 0.04	5.66
EPSΔphaJ2	3.53 ± 0.02	2.37 ± 0.09	32.97
EPSΔphaJ3	3.01 ± 0.09	2.15 ± 0.07	28.38
EPSΔphaJ4	2.84 ± 0.04	2.11 ± 0.05	25.76
EPSΔphaJ5	3.13 ± 0.03	2.30 ± 0.03	26.52
EPSΔ5phaJ	3.33 ± 0.03	3.06 ± 0.12	7.98
