# Supplementary material for: Enoyl-CoA hydratase mediates polyhydroxyalkanoate mobilization in Haloferax mediterranei
Source: Sci Rep. 2016 Apr 7;6:24015. doi: 10.1038/srep24015 (PMC4823750; doi:10.1038/srep24015)
Supplement: Supplementary Information [file srep24015-s1.pdf]

Supplementary Information

Enoyl-CoA hydratase mediates polyhydroxyalkanoate mobilization in *Haloferax mediterranei*

Guiming Liu[1, 2, 3], **Shuangfeng Cai**[1, †], **Jing Hou**[1, ††], **Dahe Zhao**[1, 3], **Jing Han**[1], **Jian Zhou**[1], **Hua Xiang**[1, *]

[1]State Key Laboratory of Microbial Resources, Institute of Microbiology, Chinese Academy of Sciences, Beijing, China.

[2]CAS Key Laboratory of Microbial Physiological and Metabolic Engineering, Institute of Microbiology, Chinese Academy of Sciences, Beijing, China.

[3]University of Chinese Academy of Sciences, Beijing, China.

[†]Current address: Institute of Molecular Medicine, Huaqiao University, Xiamen, China.

[††]Current address: School of Food and Biological Engineering, Jiangsu University, Zhenjiang, China

* Correspondence and requests for materials should be addressed to H.X. (email: xiangh@im.ac.cn).

Table S1. Strains and plasmids that were used in this study

Strain or plasmid	Relevant characteristics	Source or reference
Strains		
Escherichia coli JM109	*recA1 supE44 endA1 hsdR17 gyrA96 relA1 thi*	[1]
E. coli JM110	*dam*$^{-}$ *dcm*$^{-}$ of *E. coli* JM109	[2]
Haloferax mediterranei	CGMCC 1.2087 (=ATCC 33500)	CGMCC
H. mediterranei EPS	*pyrF* and *eps* deletion mutant of *H. mediterranei*	[3]
H. mediterranei EPSΔphaJ1	*phaJ1* deletion mutant of *H. mediterranei* EPS	This study
H. mediterranei EPSΔphaJ2	*phaJ2* deletion mutant of *H. mediterranei* EPS	This study
H. mediterranei EPSΔphaJ3	*phaJ3* deletion mutant of *H. mediterranei* EPS	This study
H. mediterranei EPSΔphaJ4	*phaJ4* deletion mutant of *H. mediterranei* EPS	This study
H. mediterranei EPSΔphaJ5	*phaJ5* deletion mutant of *H. mediterranei* EPS	This study
H. mediterranei EPSΔ5phaJ	five *phaJs* deletion mutant of *H. mediterranei* EPS	This study
H. mediterranei EPSΔ2phaB	two *phaBs* deletion mutant of *H. mediterranei* EPS	This study
H. mediterranei EPSΔ5J2B	two *phaBs* deletion mutant of *H. mediterranei* EPSΔ5phaJ	This study
Haloferax volcanii H1424	Δ*pyrE2* Δ*hdrB* pitANph Δ*mrr*, *cdc48d* c-terminal truncation	[4]
Plasmids		
pHFX	4.0 kb, vector (*pyrF*-marker) for gene knockout; Amp^{r}	[5]
pDJ1	5.1 kb, integration vector of pHFX for *phaJ1* knockout	This study
pDJ2	4.8 kb, integration vector of pHFX for *phaJ2* knockout	This study
pDJ3	4.7 kb, integration vector of pHFX for *phaJ3* knockout	This study
pDJ4	5.1 kb, integration vector of pHFX for *phaJ4* knockout	This study
pDJ5	5.1 kb, integration vector of pHFX for *phaJ5* knockout	This study
pDB1	5.6 kb, integration vector of pHFX for *phaB1* knockout	This study
pDB2	5.6 kb, integration vector of pHFX for *phaB2* knockout	This study
pTA05	8.0 kb, expressing plasmid with N-terminal His_6 tag	[6]
pTA05-phaJ1	8.7 kb, expressing plasmid for PhaJ1	This study
pWL502	7.9 kb, shuttle vector with *pyrF*-marker; Amp^{r}	[7]
pWLJ1	8.7 kb, expressing plasmid for *phaJ1* with its promoter region	This study
pWLJ2	8.4 kb, expressing plasmid for *phaJ2* with its promoter region	This study
pWLJ3	8.4 kb, expressing plasmid for *phaJ3* with its promoter region	This study
pWLJ4	8.5 kb, expressing plasmid for *phaJ4* with its promoter region	This study
pWLJ5	9.0 kb, expressing plasmid for *phaJ5* with its promoter region	This study

Table S2. Primers that were used in this study

Gene	Primer[a]	Sequence (5'→3')[b]
phaJ1	dJ1F1	TATGGTACCCGAATGTAGCCTGGAGC
	dJ1R1	TATGGATCCGGCGGAATAGACCGAGTA
	dJ1F2	TATGGATCCTGCACCCGCCCAACTT
	dJ1R2	TAGTAAGCTTCCGCTGACTCTGCTCGA
	eJ1F	AGTGGTACCGTTCCCTTCGACACCATT
	eJ1R	GTAGGATCCGTTCCCACATCCAGCAAA
phaJ2	dJ2F1	CGCGGATCCCTTCGACGTCGACCCAGA
	dJ2R1	CGTATCTCTCTGGGAGAGAACTGAATCTCTACTCCATATC
	dJ2F2	GATATGGAGTAGAGATTCAGTTCTCTCCCAGAGAGATACG
	dJ2R2	GGCGGTACCGAGAGCTCGCAATGTA
	eJ2F	CGCGGATCCAAAGACCCTGAGATAAAGC
	eJ2R	GGCGGTACCGAGGAGGAAGTGAAGCAA
phaJ3	dJ3F1	CGCGGATCCTTGTACTCGGGGTTGGCT
	dJ3R1	TCCCACCGCCCGACTGCCGAGCGCCTCGTTTCGTCCGGGG
	dJ3F2	CCCCGGACGAAACGAGGCGCTCGGCAGTCGGGCGGTGGGA
	dJ3R2	GGCGGTACCCCGTCCTCAATCT
	eJ3F	CGCGGATCCATAAGCGGTGGCTCAGTT
	eJ3R	CGGGGTACCTCGCTCCGACTATCCCAC
phaJ4	dJ4F1	CGCGGATCCGGCCAGTAGACGGTTTG
	dJ4R1	CGTACTGACCGGCGATTCGCACAACACCGTCACCCCCACC
	dJ4F2	GGTGGGGGTGACGGTGTTGTGCGAATCGCCGGTCAGTACG
	dJ4R2	CGGGGTACCGTTAGGAATGGCGAAGG
	eJ4F	CGCGGATCCTACCAACGCGGTCGCAAAA
	eJ4R	CGGGGTACCGGGGTGAACGGTTAGACT
phaJ5	dJ5F1	CGCGGATCCAAACACGGAGTGACCCAA
	dJ5R1	CAGCAGCCAGTGTTAGTTAAGGGGATGGAGATCAACATGA
	dJ5F2	TCATGTTGATCTCCATCCCCTTAACTAACACTGGCTGCTG
	dJ5R2	CGGGGTACCGGGTCAGGCAGTTGTTTT
	eJ5F	CGCGGATCCCACGGACGCATACTGGA
	eJ5R	CGGGGTACCGAGGCAGAACCACAGTCAA
phaB1	dB1F1	CGCGGATCCCGGTCCCGGTTCCATCTG
	dB1R1	CGCGCTACGACGGTCTGTGCAGTGAAAG
	dB1F2	GCACAGACCGTCGTAGCGCGGTCTATCT
	dB1R2	CGGGGTACCCCACACAACAGCCGTCCT
phaB2	dB2F1	CGCGGATCCTTTTGGGTAGTTCGTTGT
	dB2R1	CTCCATCCCAGCCCTCGTTATACAATCG
	dB2F2	TAACGAGGGCTGGGATGGAGTGGTGACA
	dB2R2	CGGGGTACCAACCGTCTCAGCTCTCAC
HFX_1509	1509F	AGATGACCGAGGACGAAGC
	1509R	AGAGGGTGCTCGTGTTCG
HFX_2830	2830F	CACCTCCAGCCTTTCCATC
	2830R	TCTTGCCCATCTCCTCTGC
HFX_4016	4016F	AGCGGATGGACCTCAAGAA
	4016R	TTCTTCGCCGTAAATAACCTC
HFX_6355	6355F	CGTCGGACTCCACTTCTTCA
	6355R	GATGTCGTGCGATGAGGC
7S RNA	7sF	ACTAGGTCGGGCAGTTAGG
	7sR	CGAAGGACGAGGTTTCTACG

[a]'d##' and 'e##' were used for gene deletion and expression, respectively.

[b]Sequences representing restriction sites are underlined.

Table S3. The candidate enzymes involved in β-oxidation cycle in *H. mediterranei*

Enzyme	ID of HFX_
① Acyl-CoA dehydrogenase	HFX_1152, _1209, _1450, _1490, _2726, _4014, _4018
② (S)-Enoyl-CoA hydratase	HFX_1463, _1525, _1964, _6354, _6381
③ 3-Hydroxyacyl-CoA dehydrogenase	HFX_1509, _2830, _4016, _6355
④ 3-Ketoacyl-CoA thiolase	HFX_2006, _6015, _6051, _6356, _6358

Table S4. The GenBank accession numbers of RpoB' for phylogenetic analysis.

Species	Accession No.	Species	Accession No.	Species	Accession No.
Halanaeroarchaeum sulfurireducens	AKH97000.1	*Halostagnicola larsenii*	BAH80382.1	*Halorubrum kocurii*	WP_008848690.1
Haloarcula salaria	AHB62278.1	*Haloterrigena salina*	WP_008892858.1	*Halorubrum hochstenium*	WP_008582792.1
Haloarcula japonica	EMA34348.1	*Haloterrigena limicola*	ELZ25836.1	*Halorubrum distributum*	ELZ50929.1
Haloarcula argentinensis	WP_005537130.1	*Haloterrigena thermotolerans*	ELZ11741.1	*Halorubrum coriense*	BAH80368.1
Haloarcula amylolytica	EMA25274.1	*Haloterrigena turkmenica*	BAH80387.1	*Halorubrum californiense*	WP_008444336.1
Halococcus sediminicola	AGZ63353.1	*Halopiger xanaduensis*	BAH80362.1	*Halorubrum arcis*	EMA70894.1
Halococcus thailandensis	WP_007739607.1	*Salinarchaeum sp. Harcht-Bsk1*	YP_008055444.1	*Halorubrum aidingense*	EMA65742.1
Halococcus salifodinae	WP_005045291.1	*Halobiforma nitratireducens*	EMA41652.1	*Halorubrum saccharovorum*	ELZ38384.1
Halococcus saccharolyticus	BAH80348.1	*Halobiforma lacisalsi*	EMA32042.1	*Halorubrum terrestre*	ELZ30683.1
Halapricum salinum	AHX03163.1	*Natrialba chahannaoensis*	ELZ06355.1	*Halorubrum lipolyticum*	BAH80371.1
Haladaptatus cibarius	BAN05497.1	*Natrialba aegyptia*	BAH80391.1	*Halobellus rufus*	AHG32318.1
Haladaptatus paucihalophilus	WP_007979677.1	*Natrialba taiwanensis*	WP_006826264.1	*Halolamina rubra*	AHG32321.1
Candidatus Halobonum tyrrellensis	WP_023395213.1	*Natrialba hulunbeirensis*	WP_006653591.1	*Haloquadratum sp. J07HQX50*	WP_021059525.1
Halarchaeum acidiphilum	WP_020220801.1	*Natrialba asiatica*	WP_006110583.1	*Haloquadratum walsbyi*	AEM24814.1
Haloarcula sp. CBA1115	AJF25435.1	*Natrialba magadii*	WP_012996638.1	*Halonotius sp. J07HN6*	WP_021060450.1
Haloarcula vallismortis	EMA11008.1	*Halovivax asiaticus*	ELZ12283.1	*Haloferax prahovense*	WP_008091495.1
Haloarcula sinaiiensis	BAH80412.1	*Halovivax ruber*	YP_007283233.1	*Haloferax larsenii*	WP_007544517.1
Haloarcula californiae	BAH80333.1	*Natrinema gari*	ELY78690.1	*Haloferax gibbonsii*	ELZ83819.1
Haloarcula hispanica	YP_004797428.1	*Natrinema altunense*	BAH80397.1	*Haloferax elongans*	WP_008322818.1

Haloarcula marismortui	YP_136946.1	*Natrinema versiforme*	WP_006432284.1	*Haloferax alexandrinus*	ELZ90541.1
Halococcus sp. 197A	BAN05496.1	*Natrinema pallidum*	WP_006184004.1	*Haloferax lucentense*	ELZ70765.1
Halococcus morrhuae	EMA48925.1	*Natronorubrum sulfidifaciens*	ELY46626.1	*Halogeometricum pallidum*	ELZ30049.1
Halococcus hamelinensis	EMA39891.1	*Natronorubrum bangense*	BAH80409.1	*Halogeometricum borinquense*	ELY31329.1
Halosimplex carlsbadense	ELZ22436.1	*Natronorubrum tibetense*	WP_006087691.1	*Haloferax sp. BAB2207*	ELK55036.1
Halobacterium sp. DL1	AHG02775.1	*Natronolimnobius innermongolicus*	ELY56680.1	*Haloferax sulfurifontis*	ELZ93256.1
Halobacterium salinarum	CAP15004.1	*Natronococcus jeotgali*	BAH80404.1	*Haloferax mucosum*	WP_008319747.1
Halomicrobium katesii	WP_026190135.1	*Natronococcus amylolyticus*	BAH80403.1	*Haloferax mediterranei*	EMA02653.1
Halomicrobium mukohataei	BAH80361.1	*Natronococcus occultus*	WP_015319535.1	*Haloferax denitrificans*	BAH80352.1
Natronomonas moolapensis	YP_007488631.1	*Natrinema sp. J7-2*	YP_006539540.1	*Haloferax volcanii*	ADE02778.1
Natronomonas pharaonis	YP_325719.1	*Natrinema pellirubrum*	WP_006182863.1	*Halorubrum sp. T3*	WP_017341877.1
Halorhabdus tiamatea	CCQ32816.1	*Natronobacterium gregoryi*	WP_005580592.1	*Halorubrum lacusprofundi*	WP_012659355.1
Halorhabdus utahensis	BAH80364.1	*Halorubrum halophilum*	AGZ63354.1	*Halogranum salarium*	WP_009366685.1
Halalkalicoccus jeotgali	ADJ16279.1	*Halorubrum ezzemoulense*	BAN19984.1	*Haloplanus natans*	BAH80363.1
Halophilic archaeon J07HX64	WP_021043617.1	*Halorubrum tebenquichense*	WP_006629284.1		
Haloarchaeon 3A1_DGR	WP_021075282.1	*Halorubrum litoreum*	EMA60710.1		

Supplementary references:

1 Sambrook, J. & Russell, D. W. Molecular cloning: a laboratory manual, 3rd ed. Cold Spring Harbor Laboratory Press, New York. (2001).

2 Palmer, B. R. & Marinus, M. G. The *dam* and *dcm* strains of *Escherichia coli*--a review. *Gene* **143**, 1-12 (1994).

3 Zhao, D. H. *et al.* Improving polyhydroxyalkanoate production by knocking out the genes involved in exopolysaccharide biosynthesis in *Haloferax mediterranei*. *Appl. Microbiol. Biotechnol.* **97**, 3027-3036 (2013).

4 Stroud, A., Liddell, S. & Allers, T. Genetic and biochemical identification of a novel single-stranded DNA-binding complex in *Haloferax volcanii*. *Front Microbiol* **3**, 224 (2012).

5 Liu, H. L., Han, J., Liu, X. Q., Zhou, J. & Xiang, H. Development of *pyrF*-based gene knockout systems for genome-wide manipulation of the archaea *Haloferax mediterranei* and *Haloarcula hispanica*. *J. Genet. Genomics* **38**, 261-269 (2011).

6 Liu, G. M. *et al.* A patatin-like protein associated with the polyhydroxyalkanoate (PHA) granules of *Haloferax mediterranei* acts as an efficient depolymerase in the degradation of native PHA. *Appl. Environ. Microbiol.* **81**, 3029-3038 (2015).

7 Cai, S. F. *et al.* Identification of the haloarchaeal phasin (PhaP) that functions in polyhydroxyalkanoate accumulation and granule formation in *Haloferax mediterranei*. *Appl. Environ. Microbiol.* **78**, 1946-1952 (2012).
